# Effects of Prior Microstructure on the Properties of Induction-Hardened JIS SCM440 Steel

**DOI:** 10.3390/ma18051045

**Published:** 2025-02-26

**Authors:** Shao-Quan Lu, Liu-Ho Chiu, Hsueh-Hung Cheng

**Affiliations:** Department of Mechanical and Materials Engineering, Tatung University, Taipei 104-327, Taiwan; u11001314@o365.ttu.edu.tw

**Keywords:** heat treatment, induction hardening, prior microstructure, microhardness, residual stress

## Abstract

JIS SCM440 steel is commonly used in precision parts after induction-hardening heat treatment. The fatigue behavior of induction-hardening parts largely depends on the combination of hardening depth and the magnitude and distribution of hardness and compressive residual stress. Therefore, it is necessary to determine the effects of different prior microstructures on the properties of JIS SCM440 steel after induction hardening. In the present study, the effects of prior microstructure (including spheroidized, annealed, normalized, and quenched and tempered) on the microhardness, hardening width, and residual stress of the induction-hardened specimens are investigated. The experimental results showed that the distribution behavior of residual stress in the hardened zone and heat-affected zone is due to the temperature gradient of the induction-hardening treatment. The hardened center appeared as compressive residual stress due to the martensitic transformation, which was accompanied by volume expansion. On the contrary, tensile residual stress will be generated in the heat-affected zone of incomplete phase transformation. The prior microstructure can affect the residual stress magnitude and distribution of microhardness and residual stresses due to the content of the cementite dissolved into the austenite at high temperatures. The difference in the carbon content of martensite after quenching will result in obvious differences in properties. The induction-hardened specimens with a normalized prior microstructure have the highest residual tensile stress in the heat-affected zone. The maximum residual tensile stress was 371 MPa in the heat-affected zone. The induction-hardened specimens with a quenched and tempered prior microstructure have the deepest hardening depth and widest residual compressive stress distribution range. The highest microhardness was 764 HV_0.3_, while the maximum residual compressive stress was −752 MPa.

## 1. Introduction

When selecting materials for mechanical parts, additional properties such as workability during machining, toughness in practical usage, and reasonable manufacturing costs are also considered. Low-alloy carbon steel is widely used in critical components of automobile and automation equipment such as driving shafts, crankshafts, and linear guideways. These critical components require high wear resistance and fatigue behavior to ensure their proper function. A JIS SCM440 (AISI 4140) steel can increase workability after spheroidization treatment. It is characterized by high hardness, ductility, tensile strength, and fatigue strength. In addition, it also has hardenability performance due to the addition of alloy [1,2,3]. There are many ways to improve the surface and subsurface properties of carbon steels such as heat treatments or coatings. Induction hardening is an excellent option for the fatigue behavior of parts made from JIS SCM440. It has the advantages of a relatively low cost and rapid processing time.

The induction hardening treatment of low-alloy carbon steel is often used to strengthen the surface of mechanical parts. Induction-hardening treatment can not only increase the surface hardness of mechanical parts but also affect changes in microstructures on the subsurface region. After induction hardening, the toughness of the core remains [4,5,6], whereas a higher surface hardness can provide superior wear resistance.

With induction-hardening treatment, however, the formation of a heat-affected zone will affect the properties of mechanical parts. For example, the heat-affected zone commonly presents a tensile state of residual stress and leads to a decrease in fatigue life. Research by Hu et al. [7], Areitioaurtena et al. [8], Cheenady et al. [9], and Hoa Ngan et al. [10] noted that residual tensile stress can be observed in the heat-affected zone after induction-hardening treatment. Hu et al. [7] and Gao et al. [11] studied the effect of induction quenching on fatigue life and showed that the remaining life of a damaged part with residual compressive stress is more than three times that of a part without residual compressive stress. Hayama et al. [12] studied induction hardening on low-alloy carbon steel to obtain different hardening depths and hardness. Their results show that the stability of compressive residual stress depends on hardness and that a thicker hardened layer will improve the fatigue strength. Wear resistance and fatigue behavior of induction-hardened parts depend on the correct combination of hardening depth and the magnitude and distribution of residual stresses on the surface layer. Residual compressive stress can improve the fatigue life of parts. In contrast, mechanical parts’ residual tensile stress in subsequent applications results in poor fatigue life [13,14,15].

Various processing parameters, especially induction frequency [16], power [17,18], and the shape of the induction coil [19] can affect the effectiveness of the induction-hardening treatment. For instance, the higher the induction frequency, the shallower the hardened depth [16]. In addition, the power of the induction-hardening process also affects the hardened depth and the surface residual stress [17,18]. To compare the residual stress distribution of different induction treatment parameters on the hardened zone and heat-affected zone, using X-ray diffractometry is the most widespread non-destructive method for characterizing residual stresses [20,21,22,23,24]. For induction-hardened JIS SCM440 steel, Areitioaurtena et al. [23] determined and developed a coupled multi-physical FE model to simulate induction hardening and compute the residual stress state of the cylindrical 42CrMo4 specimens along the microstructural transformations and hardness evolution. Grum [24] integrated the residual stress evolution of induction-hardened and grinding treatment. Induction surface hardening creates very desirable residual stresses in the hardened surface layer. Residual stresses are always of a compressive nature and are usually present to the depth of the induction-hardened layer. The studies investigated that the induction-surface-hardening treatment creates very desirable residual stresses in the hardened surface layer. Residual stresses are always of a compressive nature and are usually present to the depth of the induction-hardened layer.

As addressed above, verification after the induction-hardening treatment typically examines the distribution of microhardness, hardened depth, and residual stress. To date, most of the studies investigated the relationship between the induction-hardening treatment and the resulting properties (microhardness, hardened depth, and residual stress). However, there is a lack of papers addressing the effects of different prior microstructures on the microhardness and residual stress distribution from the heat-affected zone to the hardened zone of JIS SCM440 steel induction-hardened parts.

In the present study, a cylindrical JIS SCM440 specimen is the target material to be investigated. The specimens were first treated with different prior microstructures. The different prior treated specimens were then induction-hardened with different induction power levels. The microstructures, distribution of microhardness, and residual stress of the induction-hardened specimens were examined to determine the effects of the induction-hardening treatment.

## 2. Materials and Methods

### 2.1. Prior Heat Treatment of Specimens

The specimens used in the experiment were the JIS SCM440 steel, a low-alloy carbon steel manufactured by China Steel Corporation (Kaohsiung, Taiwan). The steel bar was spheroidized and machined to specimens with dimensions of 15 mm in diameter and 100 mm in length, and Table 1 lists its chemical compositions. The specimens were heated from room temperature to 860 °C (austenitizing) at a heating rate of 30 °C/min and exposed to an atmosphere with a carbon potential of 0.40 wt% for 1 h. The carbon potential was the carbon atom concentrations (provided by CO gas) in the atmosphere of the heat treatment furnace, where the concentration of carbon atoms can prevent the decarburizing phenomenon from the surface of the JIS SCM440 specimens. After the austenitizing process, the specimens were cooled from 860 °C to room temperature at different cooling rates (1.3 °C/min, 14 °C/min, and 167 °C/s) to obtain different prior microstructure as annealed, normalized, and quenched, respectively. For industrial application, the quenched specimens were tempered at 600 °C for 1 h and cooled with water before the induction hardening treatment.

The prior heat treatment was confirmed by examining surface hardness (HRB/HRC, FUTURE-TECH FR-3e, Kawasaki, Japan) with an applied load of 100 and 150 kg. Table 2 shows the surface hardness of the specimens with different prior heat treatments. The surface hardness of the spheroidized, annealed, normalized, and quenched and tempered specimens was 12 HRC, 80 HRB, 92 HRB, and 29 HRC.

### 2.2. Induction Hardening of Different Prior Treated Specimens

**Figure 1:**
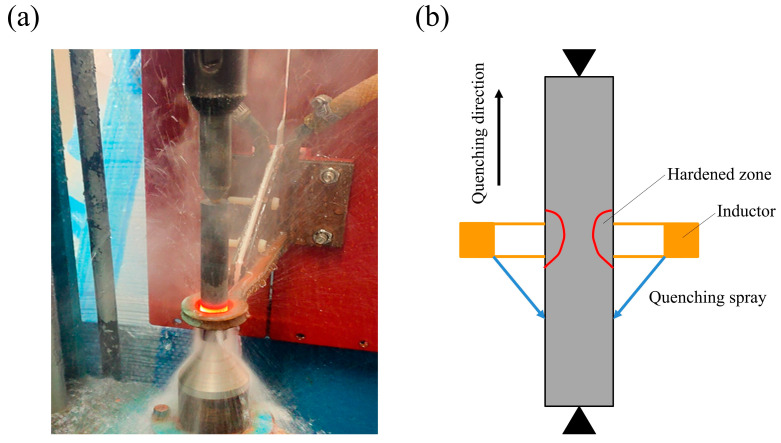
(**a**) Induction-hardening process and (**b**) schematic representation of the process setup.

The induction-hardening machine used in the experiment was a homemade system that included an inductor, induction power supply, digital control panel, controllable mobile stage, and a circled cooling water system. The frequency was set to 200 kHz, and the inductor was a single-turn coil made of copper with an internal diameter of 20 mm. The different prior treated specimens were followed by induction-hardening treatment, for which the induction power of 6, 9, and 12 kW and heated for 7 s were selected to perform induction hardening on the surface of the specimens. The specimens were kept rotating during the induction hardening to ensure uniform heating of the surface. During induction hardening, the area below the inductor is continuously quenched, while the upper area is quenched by moving the specimens downward at a rate of 25 mm/s immediately after the power has been turned off. The water was used as a quenching medium. Figure 1 shows a photograph of the induction-hardening process and a schematic representation of the process setup. All specimens were tempered at 180 °C for an hour after induction-hardening treatment to avoid cracking. Table 3 shows the induction-hardening parameters.

### 2.3. Characterizations of Induction-Hardened Specimens

After the induction-hardening treatment, the microstructure, microhardness, and residual stress of the induction-hardened specimens under the different prior microstructures were measured and addressed as follows.

#### 2.3.1. Microstructure

An abrasive wheel cutting-off machine was used to obtain the longitudinal section of specimens. After mounting, the specimens were ground to #2000 in turn and then polished with 0.05 μm alumina powder. Finally, etching was performed with 5% Nital (95 mL alcohol + 5 mL nitric acid) for microstructure observation. The differences in microstructure after prior heat treatment and induction hardening were observed with an optical microscope (OM, Olympus-BX60M, Tokyo, Japan).

#### 2.3.2. Microhardness

To determine the effect of different prior treating and induction-hardening parameters on hardness in the subsurface region, a Vickers hardness tester (Matsuzawa MXT50, Akita, Japan) was used to measure the microhardness (HV) values. To determine the microhardness distribution in the induction-hardened region, measurements were made at an interval of 1 mm at a depth of 0.2 mm from the surface. The microhardness at various depths was measured with an applied load of 300 g (2.94 N) for a duration of 10 s. A microhardness curve was established to compare the effects of induction-hardening treatment with different prior microstructures. The obtained hardness value can be used as a reference for industrial applications. It can be converted into other mechanical properties after calculation, such as tensile strength.

#### 2.3.3. X-Ray Diffraction

The surface residual stress was measured with a portable X-ray diffractometer (Pulstec μ-X360s, Pulstec Industrial Co., Ltd., Shizuoka, Japan), applying the measuring principle of the single-incident angle method (cosα method) [25,26,27]. The μ-X360s instrument used a Cr target that was operated at a voltage of 30 kV and a current of 1 mA with a diffraction angle of 35° for residual stress measurement. The X-ray diffraction parameters are listed in Table 4. The alignment of the X-ray diffractometer was confirmed by using a stress-free iron powder calibration sample prior to the experiments. Each specimen was sequentially measured from the prior microstructure area, heat-affected zone, and hardened zone to the delayed quenching area of the specimens. The presented data are average values of residual stress measured using detectors in the μ-X360s setup. A measurement error of 3–10% of the measured stress value was observed.

## 3. Results and Discussion

### 3.1. The Microstructure of Specimens Before and After Induction-Hardening Treatment

**Figure 2:**
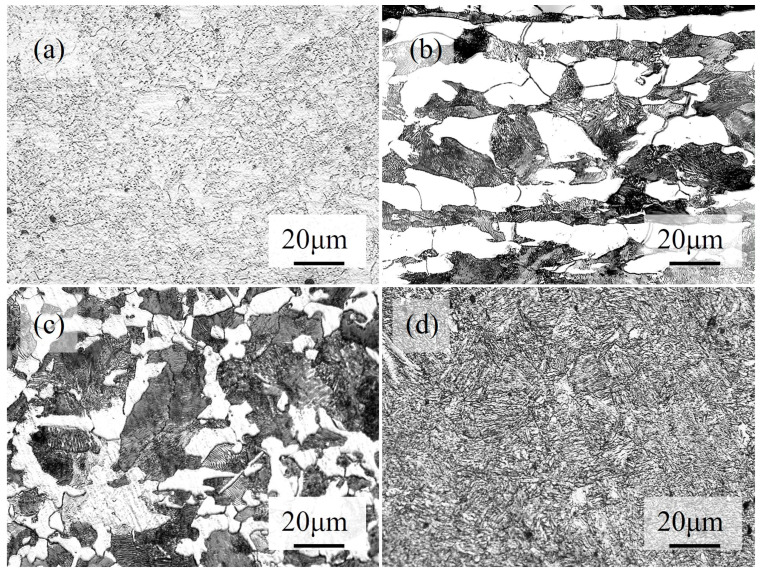
Microstructures of JIS SCM440 specimen with different prior heat treatments: (**a**) spheroidized, (**b**) annealed, (**c**) normalized, and (**d**) quenched and tempered.

Figure 2 shows the prior core microstructure of the JIS SCM440 specimens after austenitizing at 860 °C for 1 h and cooled at different rates. Figure 2a shows the core microstructure of the as-received (spheroidized) specimens: the spheroidal cementite (Fe_3_C) and granular alloy carbide dispersed in the ferrite matrix. It can be noted that the cementite after the spheroidization treatment appeared as nearly round particles, the aspect ratio of the cementite is less than a certain value, and it is called a spheroidized microstructure. Figure 2b shows the core microstructure of the specimen austenitized at 860 °C for 1 h and furnace-cooled. A coarse pearlite and isometric ferrite mixed microstructure was observed. It should be pointed out that the microstructure is neatly arranged but the grain sizes of the ferrite are different, which may come from the hot forging process of the original steel bar. Figure 2c shows the core microstructure of the specimen austenitized at 860 °C for 1 h and air-cooled. After the normalization process, the microstructure is evenly distributed rather than neatly arranged. The distribution of fine pearlite in the grain and ferrite on the grain boundaries can be observed. Figure 2d shows the core microstructure of the specimen austenitized at 860 °C for 1 h, quenched and tempered: the plate-shaped quenched and tempered martensite. The carbon atoms are dissolved in the austenite at high temperatures, where the rapid cooling leaves carbon atoms in the lattice vacancies and results in the phase transformation to martensite. With different cooling rates, the microstructure respectively changes. As reported by Bhadeshia [28], the shape and size of cementite were affected by the parameters of the heat treatment. More detailed examinations concerning the corresponding cementite’s SEM images with a higher magnification are available in Figure S1.

**Figure 3:**
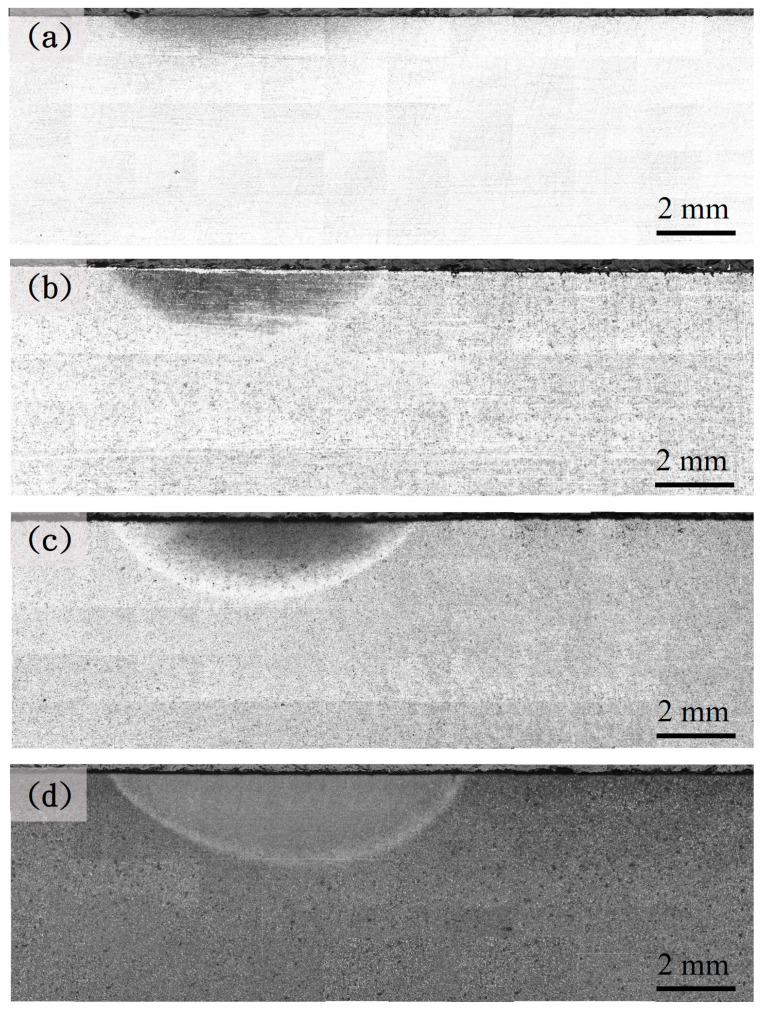
Optical microscope images of the different prior microstructure induction-hardened specimens at a power of 6 kW: (**a**) spheroidized, (**b**) annealed, (**c**) normalized, and (**d**) quenched and tempered.

**Figure 4:**
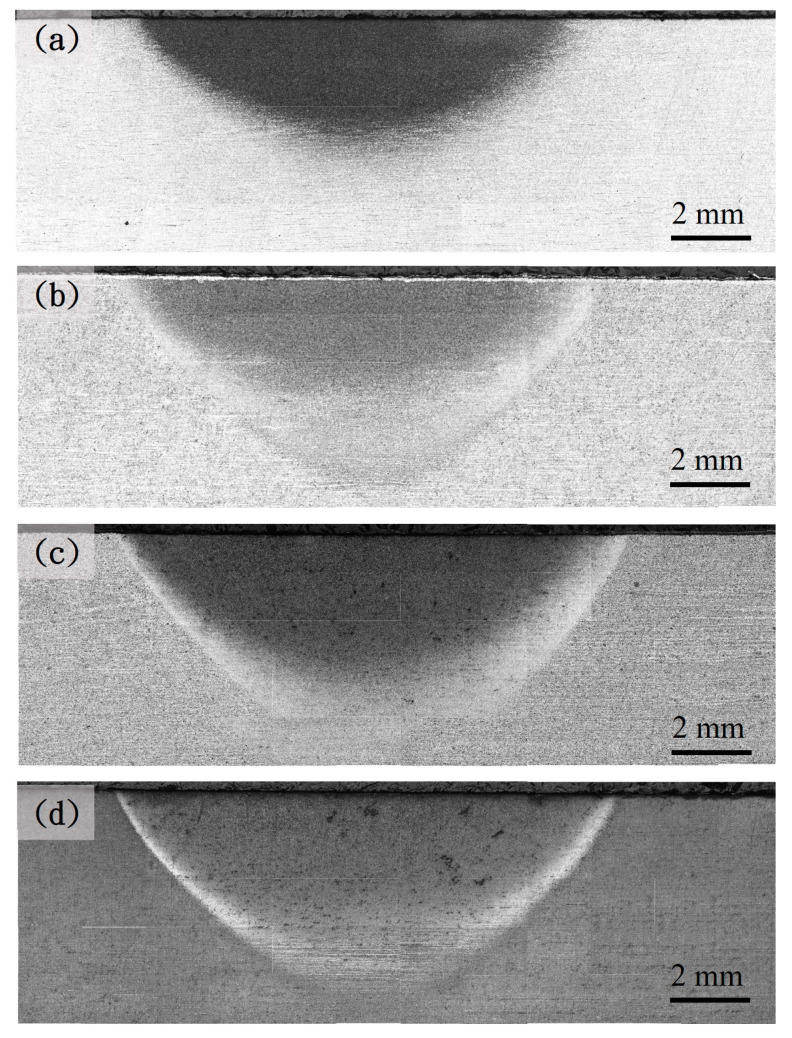
Optical microscope images of the different prior microstructure induction-hardened specimens at a power of 9 kW: (**a**) spheroidized, (**b**) annealed, (**c**) normalized, and (**d**) quenched and tempered.

**Figure 5:**
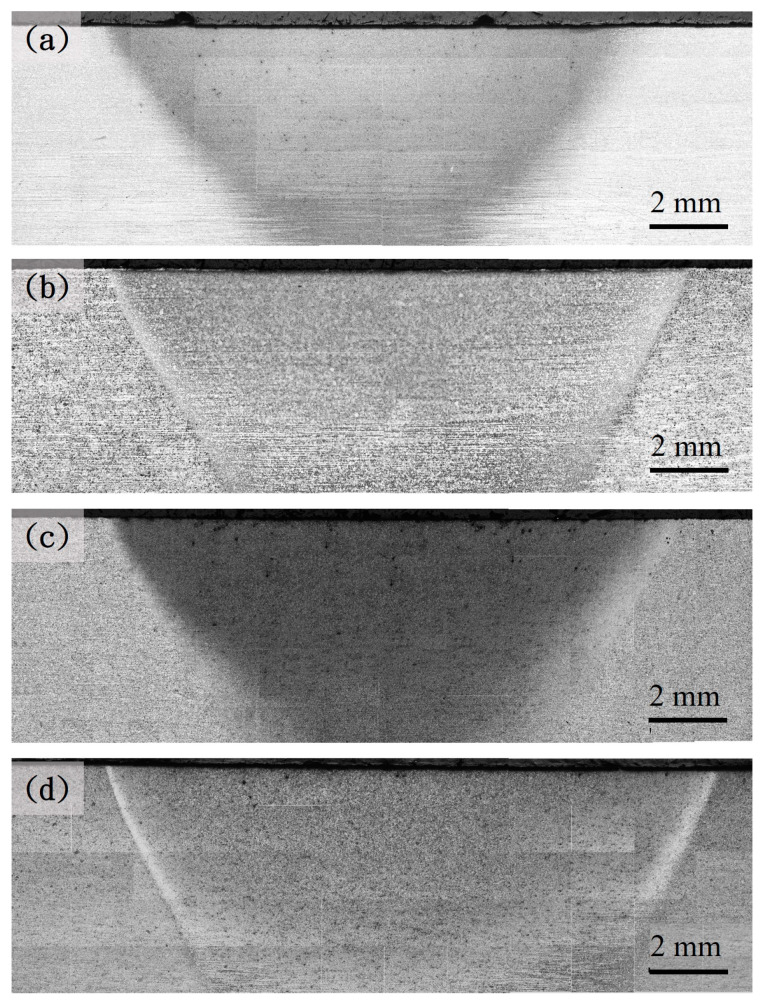
Optical microscope images of the different prior microstructure induction-hardened specimens at a power of 12 kW: (**a**) spheroidized, (**b**) annealed, (**c**) normalized, and (**d**) quenched and tempered.

Figure 3, Figure 4 and Figure 5 show the hardened region image of the different prior microstructure JIS SCM440 specimens after induction-hardening treatment at a power of 6 kW, 9 kW, and 12 kW, respectively. The increase in the hardened region can be observed with the increase in the power of the induction-hardening treatment. After the induction-hardening treatment, the length of the hardened region of the spheroidized specimens was 6 mm (Figure 3a), 10.5 mm (Figure 4a), and 13.13 mm (Figure 5a) at a power of 6 kW, 9 kW, and 12 kW, respectively. The length of the hardened zone increased with increasing induction power from 6 mm (6 kW) to 13.13 mm (12 kW), which are results similar to that reported by Hu et al. [7] and Fisk et al. [18]. The higher power of the induction-hardening treatment will increase the hardened region of the JIS SCM440 specimens. In addition, the hardened region of different prior microstructure JIS SCM440 specimens after the induction-hardening treatment at a power of 12 kW is shown in Figure 5. The length of the hardened region was 13.13 mm, 14 mm, 14.38 mm, and 15.3 mm with different prior microstructures of spheroidized, annealed, normalized, and quenched and tempered, respectively. The prior microstructure can effectively influence the length of the hardened region after induction hardening due to the size and shape of cementite (Fe_3_C). The size of Fe_3_C in quenched and tempered martensite is the smallest among all the prior states, and the induction-hardened area is the largest.

### 3.2. The Microhardness Distribution of Different Prior Microstructure Specimens After Induction-Hardening Treatment

In Section 3.1, the images of induction-hardened specimens with different prior microstructures under an optical microscope were investigated. The hardened region was increased with the induction power increase. To verify the observed image under an optical microscope and the mechanical properties of induction-hardened specimens, a Vickers hardness test with an applied load of 300 g was used. The microhardness distribution of induction-hardened specimens with different prior microstructures and induction power will be presented. Furthermore, the hardened effect of immediately quenched (negative direction from the hardening center) and delayed quenched area (positive direction from the hardening center) will also be investigated.

**Figure 6:**
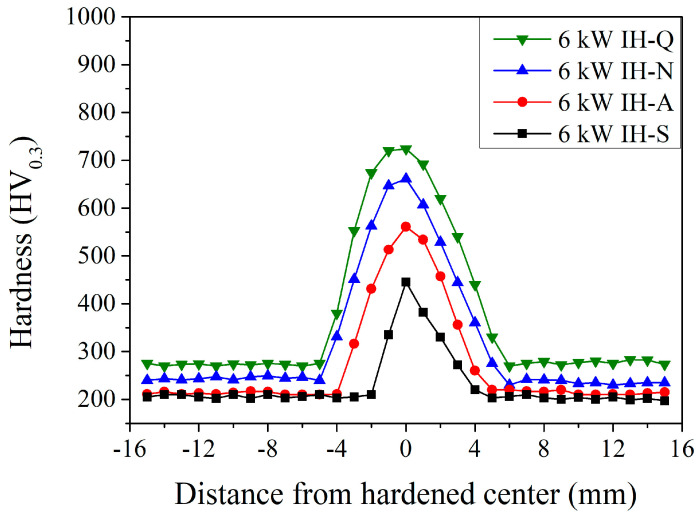
Induction-hardened microhardness specimen profiles at a power of 6 kW with different prior microstructures.

Figure 6 shows the microhardness distribution of JIS SCM440 induction-hardened specimens using a power of 6 kW with various prior microstructures (spheroidized, annealed, normalized, and quenched and tempered). In the induction-hardened specimens with a spheroidized prior microstructure (the IH-S in Figure 6), the microhardness was ~200 HV_0.3_ at a distance of −15 mm to −2 mm (unhardened area). It increased significantly to 333 HV_0.3_ at a distance of −1 mm and increased to a maximum of 445 HV_0.3_ at 0 mm (hardened center). Then, it decreased gradually to 402 HV_0.3_, 340 HV_0.3_, 271 HV_0.3_, and 200 HV_0.3_ at a distance of 1 mm, 2 mm, 3 mm, and 4 mm, respectively. The width of the hardened area was 5 mm (from −2 mm to 3 mm) and the delayed quenched area has a wider hardened effect area than the immediately quenched area. After the different prior heat treatments, the different cooling rates caused the microstructure of the specimens to transform from spheroidized to annealed, normalized, and quenched and tempered. The size of the prior spheroidized cementite (Fe_3_C) decreased, which assisted the amount of cementite solid that dissolved in the martensite matrix and that increased during the austenitizing state. As a general trend, the microhardness of the induction-hardened specimens increased with decreasing size of cementite. For the induction-hardened specimens with a prior annealed microstructure (the IH-A curve in Figure 6), the microhardness was ~215 HV_0.3_ in the unhardened area (−15 mm to −4 mm). Then, it increased significantly to 320 HV_0.3_ at a distance of −3 mm and continuously increased to a maximum of 561 HV_0.3_ at 0 mm (hardened center). Thereafter, it decreased gradually to 534 HV_0.3_, 457 HV_0.3_, 356 HV_0.3_, 265 HV_0.3_, and 216 HV_0.3_ at a distance of 1 mm, 2 mm, 3 mm, 4 mm, and 5 mm, respectively. The width of the hardened area was 8 mm (from −4 mm to 4 mm). A similar trend was observed when a normalized and quenched and tempered prior microstructure (the IH-N and IH-Q curves in Figure 6, respectively) was used. The major observed difference was the maximum microhardness at the hardened center of 0 mm and the width of the hardened area, where the microhardness was 661 HV_0.3_ and 724 HV_0.3_ and the widths were 10 mm and 11 mm with a normalized and quenched and tempered prior microstructure, respectively. The induction-hardened specimens with a quenched and tempered prior microstructure have the highest microhardness at 0 mm (hardened center) and the widest hardened area (11 mm). Similar behavior was reported by Hu et al. [7] and Gao et al. [11]. The prior microstructure will effectively affect the maximum microhardness and the hardened depth. The induction-hardened specimen with a quenched and tempered prior microstructure has the highest microhardness in the hardened case and the deepest hardened depth. The maximum microhardness increased from 445 HV_0.3_ (spheroidized) to 724 HV_0.3_ (quenched and tempered), which are results similar to those reported by Hömberg et al. [16] and Kaiser et al. [17].

**Figure 7:**
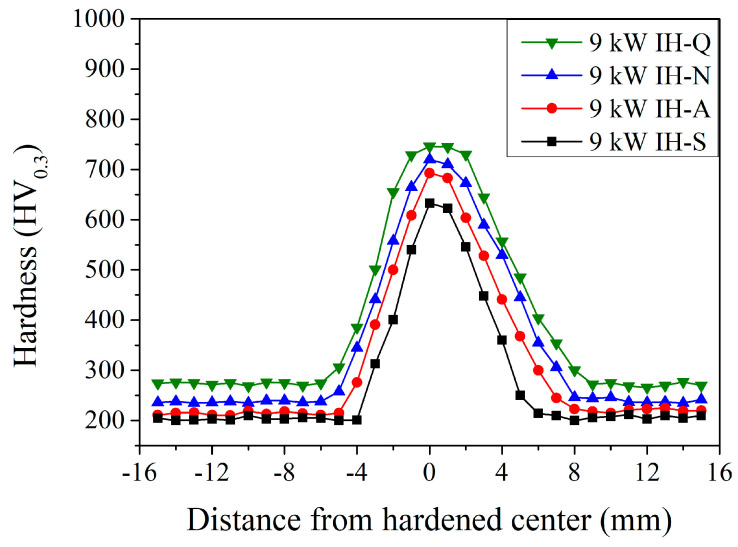
Induction-hardened microhardness specimen profiles at a power of 9 kW with different prior microstructures.

Figure 7 shows the microhardness distribution of JIS SCM440 specimens after the induction-hardening treatment using a power of 9 kW with various prior microstructures. A similar trend was observed with different prior microstructures. The microhardness and hardened area within the induction-hardened specimens increased significantly, especially with a spheroidized prior microstructure. The induction-hardened specimens with a spheroidized prior microstructure (the black curve in Figure 7), the microhardness increased significantly to 313 HV_0.3_ at a distance of −3 mm and increased to a maximum of 633 HV_0.3_ at 0 mm (hardened center). Then, it decreased to an unhardened area of 216 HV_0.3_ at a distance of 6 mm. The width of the hardened area was 9 mm (from −3 mm to 6 mm) and the delayed quenched area had a wider hardened affected area than the immediately quenched area. Thus, increasing the induction power results in a higher microhardness and a wider hardened area. At the hardened center (0 mm), the maximum microhardness was 693, 720, and 746 HV_0.3_ with an annealed, normalized, and quenched and tempered prior microstructure, respectively. The width of the hardened area was 11 mm, 12 mm, and 13 mm with an annealed, normalized, and quenched and tempered prior microstructure, respectively.

**Figure 8:**
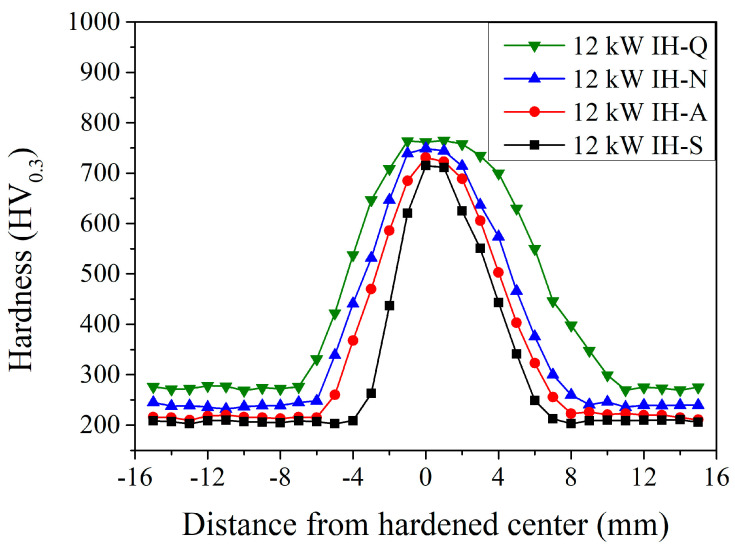
Induction-hardened microhardness specimen profiles at a power of 12 kW with different prior microstructures.

**Figure 9:**
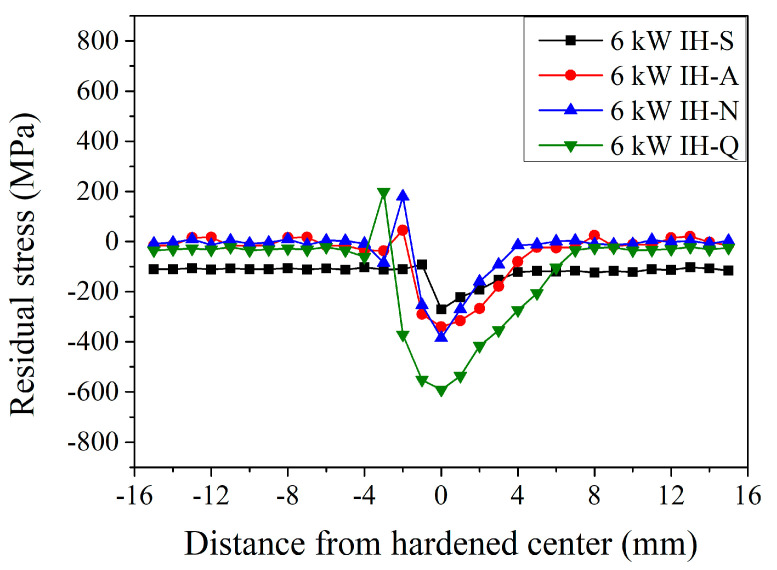
Induction-hardened surface residual stress specimen profiles at a power of 6 kW with different prior microstructures.

Figure 8 shows the microhardness distribution of JIS SCM440 specimens after the induction-hardening treatment using a power of 12 kW with various prior microstructures. It can be noted that, for the hardened center, the higher the induction power, the higher the maximum microhardness and the wider the hardened area. At the hardened center (0 mm), the maximum microhardness was 715, 731, 749, and 764 HV_0.3_ with a spheroidized, annealed, normalized, and quenched and tempered prior microstructure, respectively. Compared to those with a quenched and tempered prior microstructure (the green symbols in Figure 6, Figure 7, Figure 8 and Figure 9), the maximum microhardness increased from 720 HV_0.3_ to 762 HV_0.3_ when treated with an increased induction power. Furthermore, the width of the hardened area increased from 11 mm to 16 mm, as reported by Hömberg et al. [16] and Kaiser et al. [17]. The increased induction power will increase the maximum microhardness and the range of the hardened area of the induction-hardened specimens.

### 3.3. The Surface Residual Stress Distribution of Different Prior Microstructure Specimens After Induction-Hardening Treatment

In Section 3.2, the effects of prior microstructure and induction power on the microhardness of induction-hardened specimens were investigated. The specimen with a quenched and tempered prior microstructure presents the highest maximum microhardness and the widest hardened area. The higher the induction power, the higher the microhardness and the wider the hardened area.

To further examine the condition of surface residual stress, a portable X-ray diffractometer, applying the measuring principle of the single-incident angle method (cosα method) was used [25,26,27]. As shown by the IH-S curve in Figure 9, the residual stress on the surface of the specimen with a spheroidized prior microstructure was −110 ± 10 MPa. After the induction hardening treatment with an induction power of 6 kW, the residual stress changed to −91 MPa at a distance of −1 mm and significantly increased to −271 MPa at the hardened center. Then, it significantly decreased to −222, −192, −153, −126, and −109 MPa at a distance of 1, 2, 3, 4, and 5 mm, respectively. For the induction-hardened specimens with an annealed prior microstructure (the IH-A in Figure 9), the residual stress was 0 ± 12 MPa at a distance of −15 mm to −4 mm (unhardened area). After the induction hardening treatment with an induction power of 6 kW, the residual stress state changed from unstressed to a compressive stress of −37 MPa at a distance of −3 mm and changed from a compressive stress to a tensile stress of 64 MPa at a distance of −2 mm (heat-affected zone). Then, the residual stress state changed again into a compressive stress of −290 MPa at a distance of −1 mm and decreased to a maximum compressive stress of −340 MPa at the hardened center. It increased continuously to −316, −267, −179, and −79 MPa at a distance of 1, 2, 3, and 4 mm, respectively. Thereafter, it slightly decreased to an unstressed state of −5 MPa at a distance of 5 mm. A similar trend was observed with a normalized and quenched and tempered prior microstructure (the IH-N and IH-Q curves in Figure 9, respectively). A major difference was observed at the hardened center, where the maximum residual compressive stress was −271, −340, −384, and –591 MPa with a spheroidized, annealed, normalized, and quenched and tempered prior microstructure, respectively. Moreover, the distance exhibiting residual tensile stresses depends on the width of the hardened area and the prior microstructure. The residual tensile stress of the heat-affected zone was 46, 180, and 198 MPa with an annealed, normalized, and quenched and tempered prior microstructure, respectively. This shows a similar behavior to that reported by Shojaee et al. [29]. This distribution behavior of residual stress in the hardened zone and heat-affected zone is due to the temperature gradient of the local heat treatment. It should be pointed out that the hardened center appeared as compressive residual stress due to the martensitic transformation, which was accompanied by volume expansion. On the contrary, tensile residual stress will be generated in the heat-affected zone of incomplete phase transformation and plastic distortion from the volume expansion of martensite. However, the specimen with a spheroidized microstructure did not present tensile stress in the heat-affected zone after the induction-hardening treatment. Before induction-hardening treatment, the specimen with a spheroidized microstructure has a certain degree of residual compressive stress. That may be a key factor in the absence of tensile stress in the heat-affected zone after induction-hardening treatment. In addition, the difference in residual stress for different prior microstructures may come from the size of the cementite and the effect of dissolving into the austenite at high temperatures. It can be noted that the smaller the size of the cementite, the more it will dissolve into the austenite at high temperatures. The martensite will show different degrees of lattice distortion depending on the amount of carbon atoms dissolved into the austenite. Therefore, after induction-hardening treatment, there will be differences in the specimens with different prior microstructures.

The width of the affected area with stress changes, where the width was 5, 8, 7, and 11 mm with a spheroidized, annealed, normalized, and quenched and tempered prior microstructure, respectively. The induction-hardened specimens with a quenched and tempered prior microstructure have the highest surface residual compressive stress at the hardened center and widest stress-changed area (11 mm), with a similar behavior observed in the results of the microhardness distribution. Induction hardening effectively distributes the residual compressive stress on the surface of the hardened area [7,11]. This shows a similar trend as reported by Li et al. [13] and Huang et al. [19]. The maximum residual compressive stress generated at the hardened center, however, was affected by the prior microstructure. The maximum stress was −591 MPa with a quenched and tempered prior microstructure, which are results similar to those reported by Desisa et al. [22].

**Figure 10:**
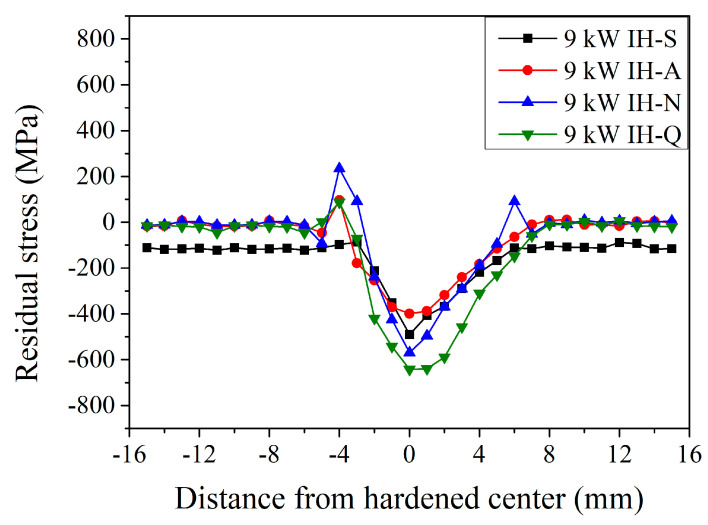
Induction-hardened surface residual stress specimen profiles at a power of 9 kW with different prior microstructures.

Figure 10 shows the surface residual stress distribution of the JIS SCM440 specimens after the induction-hardening treatment using a power of 9 kW with various prior microstructures. A similar trend was observed with a higher induction power of 9 kW. Residual compressive stress within the induction-hardened specimen increased significantly, especially at the hardened center. With a spheroidized prior microstructure (the black curve in Figure 10), the surface residual stress was −110 ± 10 MPa at an unhardened area and significantly changed to a compressive stress of −213 MPa at a distance of −2 mm after induction hardening at a power of 9 kW. It decreased to a maximum compressive residual stress of −490 MPa at a hardened center and increased continuously to −407, −368, −290, −219, and −168 MPa at a distance of 1, 2, 3, 4, and 5 mm, respectively. The residual stress was −112 MPa at a depth of 6 mm. Thus, increasing the power of the induction-hardening treatment results in a higher residual stress. At the hardened center, the residual compressive stress was −490, −399, −569, and –642 MPa of specimen induction hardened at a power of 9 kW with a spheroidized, annealed, normalized, and quenched and tempered prior microstructure, respectively. Furthermore, the width of the stress-changed area was 9, 13, 13, and 14 mm with a spheroidized, annealed, normalized, and quenched and tempered prior microstructure, respectively. In contrast, it is interesting to note that the residual tensile stress of the heat-affected zone was 97, 234, and 88 MPa with an annealed, normalized, and quenched and tempered prior microstructure, respectively. The tensile stress in the heat-affected zone of annealed and normalized specimens increased after the induction-hardening treatment. However, the tensile stress of the quenched and tempered specimen decreased after the induction-hardening treatment. Similar behavior was reported by Areitioaurtena et al. [5,8,23] and Kaiser et al. [17]. The increased power of the induction-hardening treatment will increase the residual compressive stress on the surface of the hardened center.

**Figure 11:**
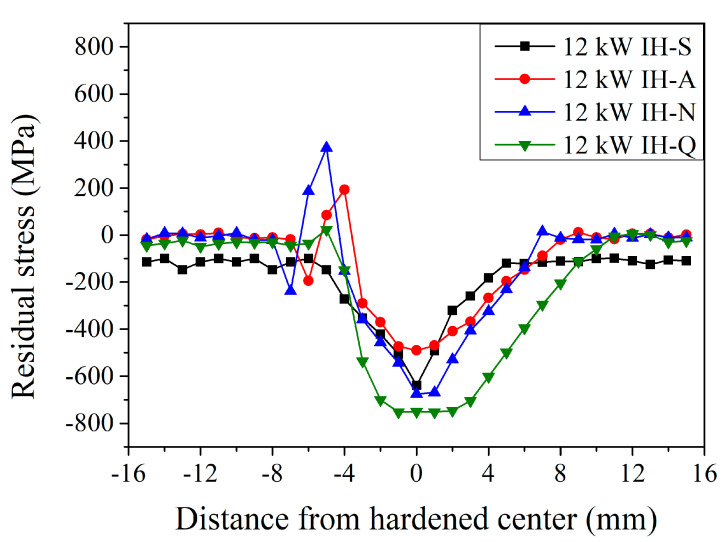
Induction-hardened surface residual stress specimen profiles at a power of 12 kW with different prior microstructures.

Figure 11 shows the results of surface residual stress distribution of JIS SCM440 specimens after the induction-hardening treatment using a power of 12 kW with various prior microstructures. Similar behavior can be observed after the induction-hardening treatment using a power of 12 kW with various prior microstructures. It is interesting to note that the maximum residual stress was −639, −491, −676, and −750 MPa when the prior microstructure was spheroidized, annealed, normalized, and quenched and tempered, respectively. The maximum residual tensile stress of the heat-affected zone was 193, 371, and 23 MPa with an annealed, normalized, and quenched and tempered prior microstructure, respectively. Moreover, the width of the stress changes area was 11, 15, 15, and 18 mm with a spheroidized, annealed, normalized, and quenched and tempered prior microstructure, respectively. Compared to those with a quenched and tempered prior microstructure (the green symbols in Figure 9, Figure 10 and Figure 11), the maximum residual compressive stress increased from −591 MPa to −750 MPa when treated with an increased induction power. Furthermore, the width of the stress-changed area increased from 11 mm to 18 mm. This shows a similar trend to that reported by Li et al. [13]. The higher the energy, the higher the residual tensile stress, and the wider the width of the stress-changed zone due to the increase in induced power.

## 4. Conclusions

In the present study, specimens with different prior microstructures underwent induction-hardening treatment using induction power of 6, 9, and 12 kW. The distribution of microhardness and residual stress was investigated and the following conclusions were drawn:

1. The prior microstructure can effectively influence the hardened region after induction-hardening treatment, and the difference in the cooling rate caused the delayed quenched area to have a wider hardened affected area than that of the immediately quenched area.
2. The distribution of residual stress in the hardened zone and heat-affected zone is due to the temperature gradient of the induction-hardening treatment. The hardened center appeared as compressive residual stress due to the martensitic transformation, which was accompanied by volume expansion. On the contrary, tensile residual stress will be generated in the heat-affected zone of incomplete phase transformation and plastic distortion.
3. The prior microstructure will effectively affect the maximum microhardness and the width of the hardened area. The smaller size of the cementite in the prior microstructure will increase the amount of the cementite that dissolved into the austenite at high temperatures. This resulted in an increase in the carbon content of the martensite and increased the hardness and width of the hardened area. The induction-hardened specimen with a quenched and tempered prior microstructure has the highest microhardness at the hardened center and the widest hardened area. The maximum microhardness and the hardened width of the induction-hardened specimen with a quenched and tempered prior microstructure were 762 HV_0.3_ and 16 mm after induction-hardening treatment at a power of 12 kW.
4. Induction hardening effectively distributes the residual compressive stress on the surface of the hardened area and is affected by the prior microstructure. The specimen with a quenched and tempered prior microstructure after induction hardening treatment at a power of 12 kW resulted in the optimal effect. The maximum residual stress reached −750 MPa (at the hardened center) and the stress-changed area was also the widest (18 mm). Moreover, the presented residual tensile stress was only 23 MPa.

## Figures and Tables

**Table 1:** Chemical compositions (wt.%) of the JIS SCM440 steel.

	C	Si	Mn	P	S	Cr *	Ni	Mo*	Cu	Fe.
JIS SCM440 Spec.	0.38–0.43	0.15–0.35	0.60–0.85	≤0.030	≤0.030	0.90–1.20	≤0.25	0.15–0.30	≤0.30	Bal.
Specimens	0.415	0.214	0.663	0.0099	0.0108	0.925	0.0473	0.164	0.101	Bal.

* For the JIS SCM440 steel, the added chromium provides good hardenability, and the molybdenum provides hardness uniformity and higher strength than carbon steel.

**Table 2:** Surface hardness of different prior treated JIS SCM440 specimens.

Surface Hardness	
**Prior Heat Treatment**	
Spheroidized (S)	12 HRC
Annealed (A)	80 HRB
Normalized (N)	92 HRB
Quenched and Tempered (Q)	29 HRC

**Table 3:** Induction-hardening process parameters.

Sample	Power (kW)	Prior Treat *
1	6	S
2	9	
3	12	
4	6	A
5	9	
6	12	
7	6	N
8	9	
9	12	
10	6	Q
11	9	
12	12	

* The induction-hardening parameters were the same for these tests. The prior treats of spheroidized, annealed, normalized, and quenched and tempered are coded as S, A, N, and Q, respectively.

**Table 4:** The X-ray diffractometer parameters.

Diffractometer Parameters	Specification/Values
Tube type	Cr (λ = 0.229093 nm)
Diffraction plane (h, k, l)	αFe(211)
Bragg angle for diffraction (2θ)	156.5°
Current	1.5 mA
Voltage	30 kV
Exposure time	15 s
Collimator diameter	1 mm
Collimator distance	51 mm
Tube type	Cr (λ = 0.229093 nm)

## Data Availability

The original contributions presented in this study are included in the article/Supplementary Material. Further inquiries can be directed to the corresponding authors.

## Supplementary Materials

The following supporting information can be downloaded at: https://www.mdpi.com/article/10.3390/ma18051045/s1, Figure S1: Cementite images of the JIS SCM440 specimens cooling at different rates after the austenitization treatment (a) spheroidized, (b) annealed, (c) normalized and (d) quenched and tempered.
