# Supplementary material for: Effects of Prior Microstructure on the Properties of Induction-Hardened JIS SCM440 Steel
Source: Materials (Basel). 2025 Feb 26;18(5):1045. doi: 10.3390/ma18051045 (PMC11901106; doi:10.3390/ma18051045)
Supplement: Supplementary file 1 [file materials-18-01045-s001.zip › materials-3464176-supplementary.pdf]

Figure S1 shows the microstructure (cementite) of the JIS SCM440 specimens after austenitizing at 860 °C for 1 h and cooling at different rates. The shape and size of the cementite were affected by the parameters (cooling rate) of the heat treatment. The microstructure in a higher magnification shows that the cementite's shape appeared nearly spherical after spheroidization treatment. The cementite appears in coarser and finer layers, after annealing treatment and normalizing treatment, respectively. After quenching and tempering, the small cementite cannot be effectively observed because it was dispersed into the tempered martensite matrix.

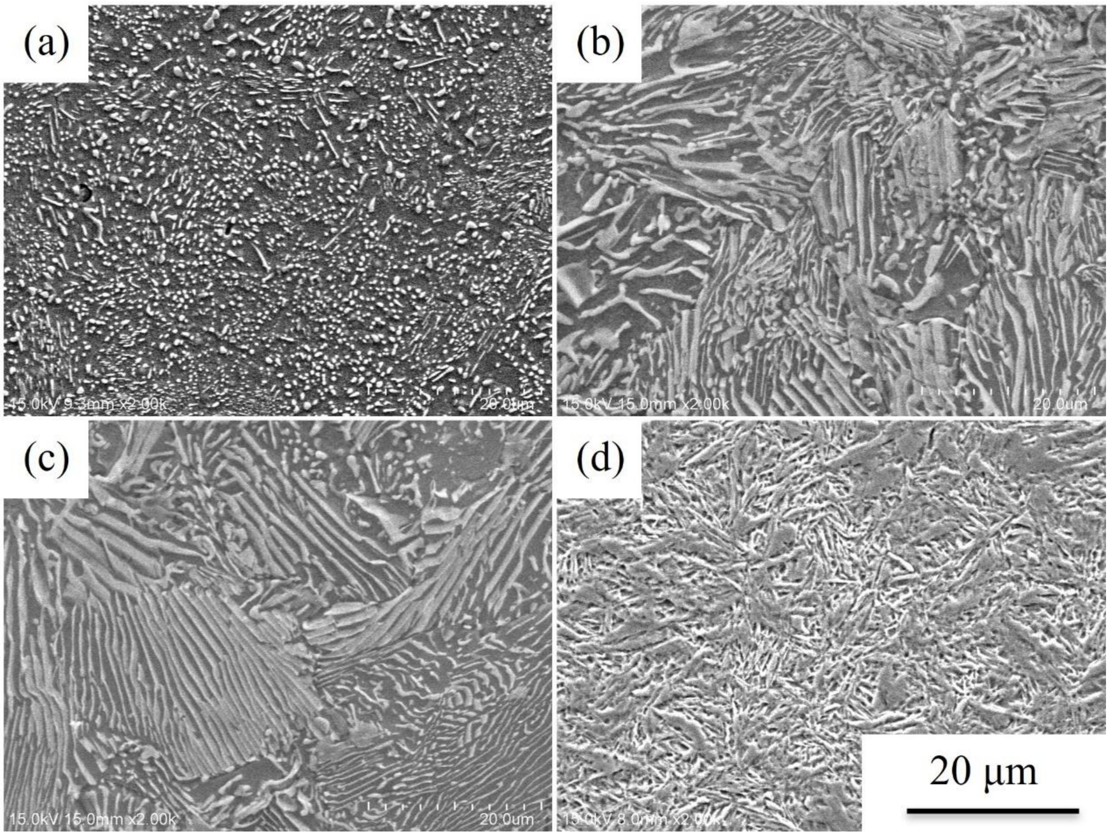

Figure S1: Cementite images of the JIS SCM440 specimens cooling at different rates after the austenitization treatment: (a) spheroidized, (b) annealed, (c) normalized, and (d) quenched and tempered.
